# Absence of Nuclear p16 Is a Diagnostic and Independent Prognostic Biomarker in Squamous Cell Carcinoma of the Cervix

**DOI:** 10.3390/ijms21062125

**Published:** 2020-03-19

**Authors:** Saioa Mendaza, Joaquín Fernández-Irigoyen, Enrique Santamaría, Tamara Zudaire, Rosa Guarch, David Guerrero-Setas, August Vidal, José Santos-Salas, Xavier Matias-Guiu, Karina Ausín, María José Díaz de Cerio, Esperanza Martín-Sánchez

**Affiliations:** 1 Molecular Pathology of Cancer Group, Navarrabiomed, Complejo Hospitalario de Navarra (CHN), Universidad Pública de Navarra (UPNA), Instituto de Investigación Sanitaria de Navarra (IdiSNA), Irunlarrea 3, 31008 Pamplona, Spain; 2 Proteored-ISCIII, Proteomics Unit, Navarrabiomed, Complejo Hospitalario de Navarra (CHN), Universidad Pública de Navarra (UPNA), Instituto de Investigación Sanitaria de Navarra (IdiSNA), Irunlarrea 3, 31008 Pamplona, Spain; 3 Department of Pathology, Complejo Hospitalario de Navarra (CHN), Irunlarrea 3, 31008 Pamplona, Spain; 4 Department of Pathology, Hospital Universitari de Bellvitge, IDIBELL, Carrer de la Feixa Llarga, 08907 L’Hospitalet de Llobregat, Spain; 5 Department of Pathology, Complejo Asistencial Universitario, Altos de Nava, 24071 León, Spain; 6 Department of Pathology and Molecular Genetics, Hospital Universitari Arnau de Vilanova, University of Lleida, Alcalde Rovira Roure 80, 25198 Lleida, Spain

**Keywords:** cytoplasmic p16, nuclear p16, subcellular location, predictive biomarker, squamous cell carcinoma of the cervix, high-grade squamous intraepithelial lesion, cervical cancer

## Abstract

The tumor-suppressor protein p16 is paradoxically overexpressed in cervical cancer (CC). Despite its potential as a biomarker, its clinical value and the reasons for its failure in tumor suppression remain unclear. Our purpose was to determine p16 clinical and biological significance in CC. p16 expression pattern was examined by immunohistochemistry in 78 CC cases (high-grade squamous intraepithelial lesions (HSILs) and squamous cell carcinomas of the cervix –SCCCs). CC cell proliferation and invasion were monitored by real-time cell analysis and Transwell^®^ invasion assay, respectively. Cytoplasmic p16 interactors were identified from immunoprecipitated extracts by liquid chromatography-tandem mass spectrometry, and colocalization was confirmed by double-immunofluorescence. We observed that SCCCs showed significantly more cytoplasmic than nuclear p16 expression than HSILs. Importantly, nuclear p16 absence significantly predicted poor outcome in SCCC patients irrespective of other clinical parameters. Moreover, we demonstrated that cytoplasmic p16 interacted with CDK4 and other unreported proteins, such as BANF1, AKAP8 and AGTRAP, which could sequester p16 to avoid nuclear translocation, and then, impair its anti-tumor function. Our results suggest that the absence of nuclear p16 could be a diagnostic biomarker between HSIL and SCCC, and an independent prognostic biomarker in SCCC; and explain why p16 overexpression fails to stop CC growth.

## 1. Introduction

Squamous cell carcinoma of the cervix (SCCC) accounts for 70–80% of all cervical cancer (CC) cases, which is the fourth most frequent cancer type in women worldwide [1], with a 5-year survival of 57–67% in Europe [2]. SCCC pathogenesis is a slow multi-stage process: when a high-risk human papillomavirus (HR-HPV) infection—present in 99.7% of cases [2,3]—persists in dividing cells, these can originate a high-grade squamous intraepithelial lesion (HSIL); if, additionally, some molecular alterations occur, tumor cells extend beyond the basement membrane of the epithelium, and then an invasive carcinoma appears [3,4]. Unfortunately, early SCCC is often asymptomatic and detected at an advanced stage when treatments are ineffective. Developments in early diagnosis are therefore much needed [1]. Currently, the standard method for SCCC diagnosis has many limitations because it is mainly based on subjective interpretation of histological changes in cervical tissue [2,4,5,6]. Intriguingly, as CC development seems to be a continuous process, it is quite difficult to morphologically distinguish an HSIL which has begun to invade and an SCCC which has just penetrated the basement membrane. However, these apparently similar tumors display very different clinical behaviors. Women with HSIL are treated with surgery that is usually successful, with more than 85% of patients being cured [7]; those with SCCC receive surgery plus chemoradiotherapy, but unfortunately, half of them die from the disease [2]. An accurate diagnosis is therefore crucial for the successful clinical management of these patients. 

Recently, an HPV DNA test has been introduced as a screening tool; this has proved to be much more sensitive than traditional methods [2], indicating that molecular biomarkers are very informative and of great utility. Indeed, they are objective, quantitative and easily reproducible in all hospitals and laboratories, for many tumor types [8,9,10]. However, to date, no molecular panel has been available for SCCC, in contrast to other malignancies, such as breast [8,11], lung [12] and colorectal [13] cancers, so there is an urgent need for molecular biomarkers that will enable early diagnosis, prognosis and targeted therapy for SCCC [14]. A better understanding of the molecular mechanisms leading to SCCC could help us develop better clinical strategies to improve patients’ likelihood of survival. In fact, the European Society of Medical Oncology has recommended that more research be undertaken to identify molecular biomarkers in CC [2].

The cyclin-dependent kinase inhibitor 2A protein, also called p16, is a well-known tumor-suppressor protein which is down-regulated in many tumors, and is involved in regulating cell cycle, senescence, apoptosis, cell invasion and angiogenesis [3,5,15]. In spite of these well-recognized tumor-suppressor properties, p16 expression paradoxically increases with the severity of the cervical lesion [3,5,14,16], and has been then proposed as a useful marker for identifying such lesions [5,17,18,19,20]. In fact, its ancillary use in cervical cytology has improved the accuracy of histological diagnosis [5,21], mainly in premalignant lesions [4]. However, the specificity of p16 has been insufficiently studied in SCCC [18], and its prognostic value remains controversial [3,5,14].

As there is a lack of consensus and clear guidelines regarding the use of p16 expression in routine clinical management of SCCC [2,5,19], we aimed to shed some light on the clinical utility and biological significance of p16 overexpression during SCCC progression.

## 2. Results

### 2.1. Expression Pattern and Clinical Relevance of p16 in SCCC Patients

**Figure 1:**
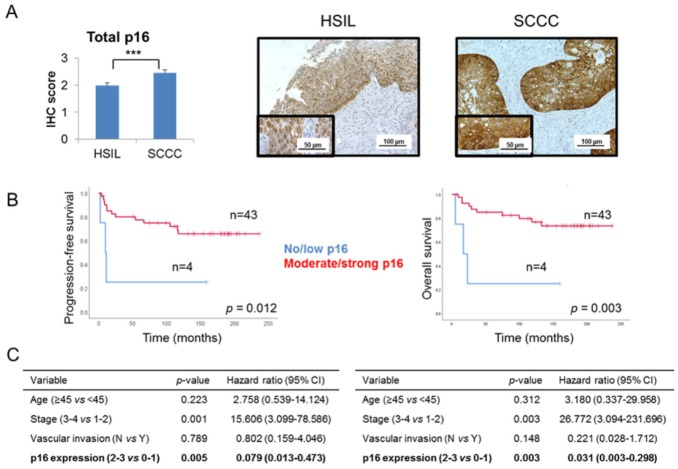
Global p16 expression in cervical tumors. (**A**) Immunohistochemical expression of p16 was examined in a series of 29 high-grade squamous intraepithelial lesions (HSILs) and 49 squamous cell carcinomas of the cervix (SCCCs) (*** *p* < 0.001). Representative images are shown at 200× magnification, and details are highlighted at 400×. (**B**) Association between global p16 expression and progression-free survival (PFS) and overall survival (OS) in SCCC patients. (**C**) Multivariate analysis showing the independent association between p16 expression and PFS (left) or OS (right), regardless of vascular invasion, stage and age of SCCC patients. (CI, confidence interval).

Global p16 expression was examined in our series of 29 HSILs and 49 SCCCs, taking into account the criteria which are routinely used in the clinical practice. We observed that SCCC tumors had a significantly higher level of p16 expression than did HSILs (*p* < 0.001) (Figure 1A and Table 1). No statistical association was found between global p16 expression and pathological variables (data not shown). Interestingly, significant associations between a high level of global p16 expression and good prognosis and outcome were found in SCCC patients (Figure 1B), independent of other clinical parameters crucial in SCCC prognosis, such as age, stage and vascular invasion (Figure 1C and Figure S1). Indeed, Cox analyses showed that strong expression of p16 had a protective effect against relapse and death (hazard ratios 0.079 and 0.031 for progression-free survival (PFS) and overall survival (OS), respectively) (Figure 1C). These results contribute to the paradox of p16 overexpression in SCCC, since a tumor-suppressor protein which significantly adds to a good outcome is overall overexpressed in this aggressive cancer type. 

**Figure 2:**
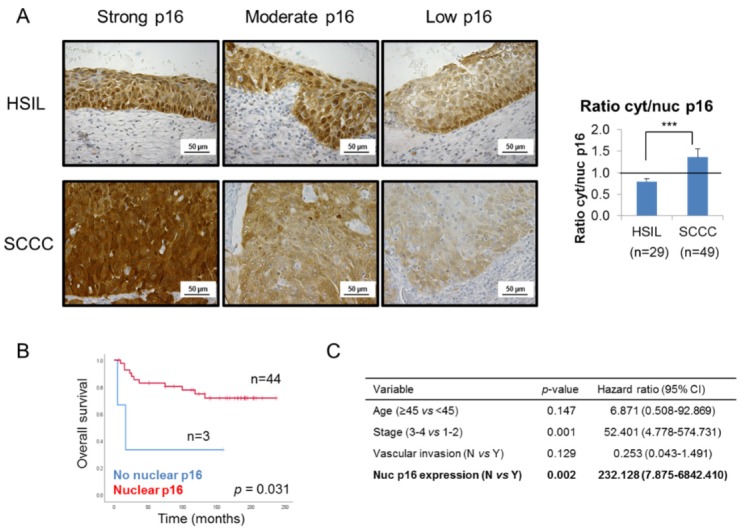
Subcellular location of p16 in cervical tumors. (**A**) Representative images of p16 staining in 3 high-grade intraepithelial lesions (HSILs) and 3 squamous carcinomas of the cervix (SCCCs) at 400× magnification showing that SCCC samples (*n* = 49) showed stronger cytoplasmic than nuclear p16 expression than HSIL (*n* = 29), regardless of the degree of global p16 staining (strong, moderate or low). Note p16 negativity in the nuclei of the low p16-expressing SCCC sample. The ratio cytoplasmic to nuclear (cyt/nuc) p16 expression was calculated by dividing p16 immunohistochemical score in each subcellular compartment. The horizontal line in the histogram shows equal amounts of cytoplasmic and nuclear p16 (ratio cyt/nuc p16 = 1) (*** *p* < 0.001). (**B**) Association between p16 subcellular location and overall survival (OS) in SCCC patients. (**C**) Multivariate analysis revealed an independent association between p16 subcellular location and OS, regardless of vascular invasion, stage and age of SCCC patients. (CI, confidence interval).

In order to shed some light on this controversy, we focused on p16 subcellular location, and observed that SCCC tumors showed stronger cytoplasmic than nuclear staining than the HSIL samples, regardless of the global expression of the protein (Figure 2A and Table 1). Indeed, SCCC samples displayed a significantly higher cytoplasmic/nuclear p16 ratio than HSIL (*p* < 0.001). This finding suggests that not only p16 expression, but also its subcellular location, could be diagnostic biomarkers which can distinguish between HSIL and SCCC. 

Furthermore, although no significant association was found between nuclear or cytoplasmic p16 expression and pathological variables (data not shown), the absence of nuclear p16, observed in only 6% of SCCC patients though (Table 1), was significantly associated with a very poor outcome (*p* = 0.031) (Figure 2B and Table S1). Moreover, this association was significantly independent of clinical parameters like age, stage or vascular invasion (*p* = 0.002); and we found that the lack of nuclear p16 significantly increased the risk of death by more than 200-fold (Figure 2C).

These findings indicate that although p16 is globally overexpressed in SCCC, it is not mainly located in the nucleus, where it could exert its tumor-suppressor functions. Therefore, these results could explain the p16 paradox in SCCC.

### 2.2. p16 Subcellular Location in SCCC Cell Lines

**Figure 3:**
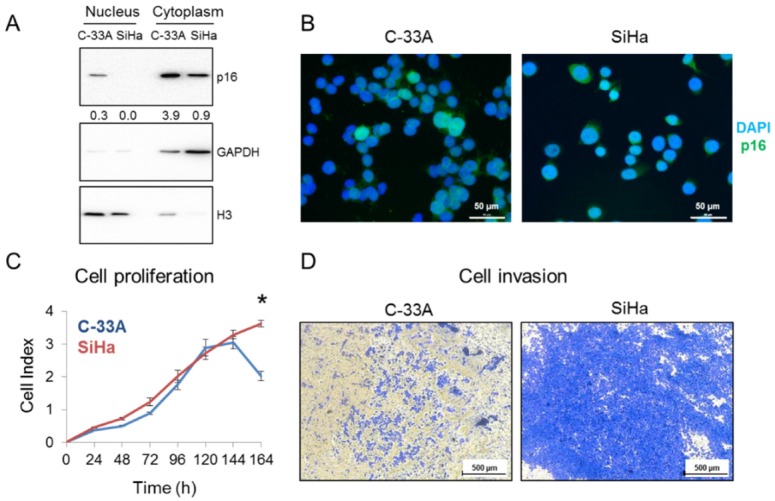
p16 subcellular location in SCCC cell lines. (**A**) Nuclear and cytoplasmic protein fractions of C-33A and SiHa were separately subjected to western blot to check p16 expression. GAPDH and Histone H3 were used as loading controls of each subcellular fraction. Numbers indicate the ratio of p16 signal relative to that in the loading control, measured by densitometry. (**B**) p16 expression was examined by immunofluorescence in C-33A and SiHa cells. Images were acquired at 400× magnification. (**C**) Cell proliferation of C-33A and SiHa cell lines was measured by real-time cell analysis for 7 days (* *p* < 0.05). (**D**) Cell invasion of C-33A and SiHa cell lines was examined by their ability to penetrate a Matrigel^®^ layer for 3 days. Images were acquired at 50× magnification.

p16 expression pattern was also interrogated in SCCC cell lines. Western blot (Figure 3A) and immunofluorescence (Figure 3B) revealed that C-33A cells expressed p16 in both the nucleus and the cytoplasm, while SiHa cells only expressed it in the cytoplasm. Similar to what we observed in SCCC tumors, this differential subcellular location could affect cell aggressiveness, as SiHa had greater proliferative (Figure 3C) and invasive (Figure 3D) properties than did C-33A. These abilities were also examined in the HeLa cell line (Figure S2), which was derived from a cervical adenocarcinoma, with nearly identical clinical features to SCCC [22]. Interestingly, we found a very similar pattern of p16 expression and growth and invasive capacities in HeLa and SiHa cells, indicating that the absence of nuclear location of p16 is related to CC aggressiveness.

### 2.3. Cytoplasmic p16 Interactome in SCCC

**Figure 4:**
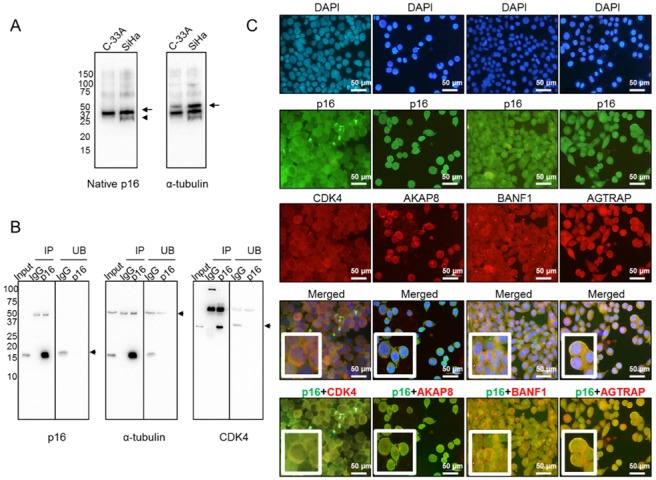
Cytoplasmic p16 interactors in SiHa cells. (**A**) Native form of p16 protein was examined in both C-33A and SiHa cell lines by western blot under non-denaturing conditions. Arrows point to the proteins of interest, while the arrowhead indicates a different 3D conformation of native p16 in SiHa, as compared with C-33A. α-tubulin was used as a loading control. (**B**) Exclusively cytoplasmic p16 was immunoprecipitated from SiHa cells, and efficiency was checked by western blot. Immunoprecipitated (IP) and unbound (UB) fractions upon incubation with IgG and anti-p16 antibodies are shown. α-tubulin was used as a loading control. Immunoprecipitation of p16 interactors was checked by incubating the same membrane with an anti-CDK4 antibody, a very well-known p16-interacting protein. The arrows indicate the proteins of interest. (**C**) Colocalization in the cytoplasm of SiHa cells of p16 (in green) and four interactors (in red), identified by liquid chromatography-tandem mass spectrometry (LC-MS/MS), was revealed by double immunofluorescence. Images were acquired at 400× magnification.

To understand why p16 is mainly confined to the cytoplasm in aggressive SCCC tumors, we hypothesized that it could be sequestered by other proteins to form large macromolecular complexes in order to avoid nuclear translocation, as has been described in other tumor types [15]. To test this hypothesis, we first examined the conformation of native p16 by western blot under non-denaturing conditions. We observed a band in SiHa, but not in C-33A, which may indicate a differential 3D conformation of native p16 (Figure 4A). This finding suggested that p16 could actually be bound to other proteins that sequester it in the cytoplasm. Therefore, cytoplasmic p16 was immunoprecipitated from SiHa cells, which lacked nuclear p16 (Figure 3A). Figure 4B shows immunoprecipitation efficiency regarding the pull-down of p16 and its interactors, as one of the well-known proteins that interacts with p16, the cyclin-dependent kinase 4 (CDK4) [15], was also detected in our immunoprecipitated extract (Figure 4B). To be confident in the identification of p16 interactors, those extracts were digested by taking a dual approach—in-gel and in-solution digestion—and then subjected to liquid chromatography-tandem mass spectrometry (LC-MS/MS) (Table S2). Data mining of LC-MS/MS-generated proteomic data revealed that 16 proteins were simultaneously identified in both extracts pulled down with the anti-p16 antibody, but not with IgG (Table 2). They were proposed to be at least a part of the cytoplasmic p16 interactome in SiHa cells with a false-discovery rate (FDR) < 0.01. It is worth mentioning that no protein from HPV was found to be bound to cytoplasmic p16. Importantly, we observed that the vast majority of those human interacting proteins had not been previously reported in the BioGRID interaction repository, which currently contains 229 interactors for p16 (January 2020). The only two proteins found to be common to the BioGRID list and the immunoprecipitated extracts digested in-gel and in-solution were CDK4 and C1QBP (Figure S3). Our findings therefore include 14 new potentially interacting cytoplasmic p16 proteins.

### 2.4. Colocalization of Cytoplasmic p16 and Interactors in SiHa Cells

Some of the cytoplasmic p16 interactors identified by LC-MS/MS were selected for further examination by double immunofluorescence because of their involvement in other carcinogenic processes [23,24,25,26,27,28,29]. As seen in Figure 4C, we found that CDK4, the A-kinase anchor protein 8 (AKAP8), the barrier to autointegration factor (BANF1), and the type-1 angiotensin II receptor-associated protein (AGTRAP) colocalized with p16 in the cytoplasm of SiHa cells, confirming that these proteins interact with cytoplasmic p16 in SiHa cells.

## 3. Discussion

Although p16 overexpression is a potential biomarker for CC clinical management [5,17,18], there is a lack of consensus [5,19] and standard guidelines [2] concerning its routine use. Similarly, the biological significance of the paradoxical activation of this tumor-suppressor protein in CC is not yet well understood.

We confirmed p16 overexpression in our series of 78 cervical tumors, as extensively described [3,14,16,21], since strong p16 expression is a proven useful surrogate biomarker for tumors with transcriptionally active HR-HPV [5]. Importantly, we found that p16 expression—and especially the cytoplasmic/nuclear p16 ratio—significantly distinguished HSIL from SCCC. Our findings are therefore evidence of the usefulness of p16 as a diagnostic biomarker between HSIL and SCCC, as previously proposed [18,19]. In fact, it has facilitated inter-observer agreement and then has improved the accuracy of traditional diagnostic methods based on histological observation in lower grade lesions [4,5,6,21,30]. 

Notably, a high level of p16 expression was significantly associated with longer survival in our SCCC series, irrespective of other clinical parameters such as age, stage and vascular invasion. Although some reports have associated p16 overexpression with poor prognosis [3,14], our results are consistent with those of a meta-analysis involving 1633 CC patients [5]. It is thought that HR-HPV-related tumors are less genetically altered, and therefore tend to respond better to therapy and to have improved outcome [5,15]. Here, we noticed that 6% of SCCCs had null or low p16 levels. Although our sample number is limited, we have observed the same small proportion of p16 negative SCCC patients than a recent report where such patients negative for global p16, which represented only 4% of CC cases, had significantly worse OS [31].

It has been suggested that the lack of a standardized cut-off point for p16 overexpression could have masked its true clinical value [5]. However, as it is difficult to establish a cut-off value in subjectively evaluated immunohistochemical staining, the novelty of our study relies on the relevance of p16 subcellular location. To date, p16 expression pattern remains controversial, because there is no fundamental justification yet as to whether cytoplasmic/nuclear or only nuclear staining should be taken as positive [15]. As other authors have found [5,32], there was no association between p16 expression or location and pathological variables in our study, but we did find that the lack of nuclear p16 allowed SCCC to acquire aggressive features both in tumors and *in vitro*. This interesting finding goes some way to explaining the paradoxical p16 overexpression in SCCC. It is thought that retinoblastoma (Rb) protein inhibition by HPV E7 protein triggers a negative feedback that leads to p16 overexpression, in a failed attempt to stop cell proliferation [3,5,15,17]. Our results could explain this failure, since, although overexpressed, p16 would not be able to fulfil any anti-tumor function in the cytoplasm. Cytoplasmic levels of some typical nuclear cell cycle regulators, such as p16, p27 and PTEN, have been described and associated with tumor progression in uterine leiomyosarcomas, astrocytomas, gastrointestinal stromal tumors and breast and colorectal cancer [15,17]. However, cytoplasmic p16 has been subtly examined in CC, found to be not significantly associated with the histological grade of the lesion [17] and associated with a borderline-significantly worse two-year PFS in HSIL patients (*p* = 0.049) [33].

Cytoplasmic p16 has only recently been evaluated, having been considered a background factor in many studies, and so its significance is due for reinterpretation [15]. Some authors have characterized p16 expression in CC cell lines and found very opposite results in comparison with ours: exclusively cytoplasmic p16 in C-33A cells and nuclear/cytoplasmic p16 in SiHa and HeLa cells [18]. Nevertheless, it is important to mention that they described p16 subcellular location solely in cell lines, which are just models of disease with many limitations that can influence protein levels and location. In contrast, our conclusions are supported by observations in tissues. Based on this, our study demonstrates that cytoplasmic p16 is not an artefact but has important biological and clinical implications. It has been hypothesized that the binding of p16 to other cytoplasmic proteins could form large molecules that are unable to pass through the nuclear membrane pores. Among these proteins, AE1 is thought to be present in gastric and colon cancer cells [15]; while PCNA, MCM6, α/β/γ-actin and α/β-tubulin, among others, have been observed in a lymphoblastoid cell line [34]. Although it is conceivable that cytoplasmic p16 interactors are cellular-context-dependent, CDK4 has been proposed as a strong candidate for sequestering p16 in the cytoplasm [15]. In fact, the interaction between p16 and CDK4 has already been demonstrated via co-immunoprecipitation in SiHa cells [35]. Accordingly, we also found binding and colocalization of p16 and CDK4 in SiHa cell cytoplasm, which strengthened the validity of our results. We also detected another p16 interactor already reported in the BioGRID repository, the complement component 1 Q subcomponent-binding protein (C1QBP), which has been described as associating with the *CDKN2A* product to mediate apoptosis [36]. Apart from these, 14 new interactors of cytoplasmic p16 were also identified here. Importantly, we confirmed the colocalization of p16 with a protein subset previously involved in carcinogenesis with a specific role in nuclear shuttling. For instance, BANF1 has been reported to be involved in colony formation, migration and invasion in CC [37] and in the development of hepatocellular [23] and esophageal squamous cell [24] carcinomas and gastric cancer [26]. It is also known to interact with a protein which prevents nuclear accumulation of other proteins, such as β-catenin [38]. Another novel p16 interactor identified here, AKAP8, dynamically interacts with other proteins during cell cycle progression [39] and appears to play an important role in promoting lung [27], ovarian [28] and rectal [29] cancers. Moreover, it helps deliver cyclin D/E to CDK4 [40] and interacts with a kinase which phosphorylates p27 to prevent its translocation to the nucleus [41]. Finally, we identified AGTRAP, a HIF1α direct target related to reduced PFS in melanoma [25] and to tumor growth and angiogenesis in Lewis lung carcinoma [42].

Further evaluation is warranted to confirm these observations in larger cohorts of patients, and to answer many questions that have been raised. For instance, whether p16 sequestration in the cytoplasm *per se* is the reason for the failure of its tumor-suppressor function or whether it involves more complex mechanisms is still to be determined. Since p16 overexpression has been observed at the invasive front of endometrial, colorectal and basal cell carcinoma, it has been proposed that cytoplasmic p16 is involved in the dissociation of focal adhesions, and then, related to cell invasion [15], lymphangiogenesis and lymphatic metastasis [5]. 

The potential therapeutic value of p16 is also to be elucidated. On the one hand, depletion of p16 has been shown to promote chemo- and radioresistance in HeLa cells [32]. On the other hand, it has been suggested that p16 could be a therapeutic target in CC, since its knockdown inhibits cell proliferation, migration and invasion in SiHa and HeLa cell lines [43] and sensitizes SiHa cells to cisplatin [35]. However, given the importance of its tumor-suppressor properties in other cell types, it is not clear how p16 inhibition would affect organism homeostasis. Here, we draw attention to a potential therapeutic use for p16 that works not by targeting it directly, but by inhibiting the proteins which sequester it in the cytoplasm. This strategy would be expected to release and translocate p16 to the nucleus in order to exert its tumor-suppressor function.

Recently, novel proteins, such as PDL1 [44], survivin [45] and SIRT1 [46], have become of interest as diagnostic or prognostic biomarkers in cervical malignancies. Undoubtedly, these findings are very important for SCCC clinical management, but their implementation would have economic consequences. In contrast, our results imply that it is only necessary to evaluate the p16 immunohistochemical pattern accurately—and since this is already routinely performed—it would not incur any additional cost.

## 4. Materials and Methods 

### 4.1. Patient Samples

We analyzed a series of 78 formalin-fixed, paraffin-embedded samples from women diagnosed with HSIL (*n* = 29) and SCCC (*n* = 49). All patients were diagnosed between 1995 and 2015 in the Pathology Department of the Complejo Hospitalario de Navarra (Pamplona, Spain), Hospital Universitari de Bellvitge, (L’Hospitalet de Llobregat, Spain), Complejo Asistencial Universitario (León, Spain), and Hospital Universitari Arnau de Vilanova (Lleida, Spain). Clinical characteristics of SCCC patients are summarized in Table S3. No clinical follow-up was available for HSIL patients, since they were all successfully cured by surgery. All tumors were surgically removed and staged according to their size, histological grade and lymph node involvement. All cases were ensured to harbor at least 70% tumor cells. None of the patients had received radiotherapy or chemotherapy before surgery. The study was approved by the Navarre Ethics Committee for Clinical Research (PI_2018/75) on September 30th 2019, procedures were in accordance with the Helsinki declaration, and informed consent was obtained according to the current Spanish legislation.

### 4.2. Immunohistochemistry

Sections (4-µm thick) of 29 HSILs and 49 SCCCs were placed on slides and deparaffinized. After performing antigen retrieval at 37 °C for 30 min with the cell conditioning 1 buffer (catalog number 950–124) at pH 8.4, samples were incubated with the anti-p16 antibody (catalog number 805–4713, clone E6H4) [31] at 1 µg/mL for 28 min at 37 °C in a Benchmark XT^®^ Ventana immunostainer (all reagents and equipment from Roche, Basel, Switzerland). The pattern and intensity of expression were evaluated blind by two independent observers (T.Z. and E.M.-S.), and ascribed to one of four categories: 0, no expression; 1, weak expression; 2, moderate expression; and 3, strong expression. Images were acquired with a Leica DM4000B microscope (Leica, Wetzlar, Germany) and the NIS Elements v4.30 program (Nikon Instruments, Amsterdam, Netherlands).

### 4.3. Cell Lines

Two human SCCC cell lines were used in this study, C-33A (an HPV-negative carcinoma) and SiHa (an HPV16-positive SCCC), both of which were purchased from the American Type Cell Collection (ATCC, Rockville, MD, USA). Additionally, the HeLa cell line (HPV18-positive cervical adenocarcinoma) was also obtained from the ATCC. They were all grown in DMEM supplemented with 10% fetal bovine serum (FBS) and 1% penicillin/streptomycin (all from Life Technologies, Carlsbad, CA, USA), at 37 °C in a humidified atmosphere with 5% CO_2_. Experiments were performed with *Mycoplasma*-free and recently authenticated cell lines at low passage.

### 4.4. Western Blot

p16 expression levels were checked in both cytoplasmic and nuclear fractions in three CC cell lines by western blot. Briefly, for subcellular fractionation, C-33A, SiHa and HeLa cells were grown to 70–80% confluence and lysed with a phosphate-buffered saline (PBS)-based buffer containing 1% NP-40 (Thermo Fisher Scientific, Waltham, MA, USA) and protease inhibitors (Roche, Basel, Switzerland). Cells were then scraped and centrifuged at 13,000 rpm for 1 min at 4 °C. Cytoplasmic proteins in the supernatant were collected, while pellets were resuspended in 50 μL of lysis buffer (7M urea, 2M thiourea and 50 mM DTT), incubated on ice for 30 min and sonicated for two cycles of 20 s each in a Vibra-Cell^TM^ 75185 ultrasonic liquid processor (Bioblock Scientific, Illkirch, France). Finally, samples were centrifuged at 20,000× *g* for 20 min at 15 °C, and nuclear proteins were collected from the supernatant. Protein concentrations were quantified using the Protein DC kit (Bio-Rad, Hercules, CA, USA) in an Epoch plate reader (BioTek, Winooski, VT, USA), following the manufacturers’ recommendations. For western blot, 60 μg of proteins were resolved by SDS-PAGE in a 15% polyacrylamide gel and transferred onto a nitrocellulose membrane (Bio-Rad, Hercules, CA, USA). The membrane was blocked with 5% non-fat milk and incubated with the anti-p16 antibody (805–4713, Roche, Basel, Switzerland) at 250 ng/mL overnight and at 4 °C. Then, it was incubated with the secondary anti-mouse antibody (Bio-Rad, Hercules, CA, USA) at 1:2000 for 1 h at room temperature. The signal was detected with the SuperSignal West Pico Chemiluminescent Substrate kit (Thermo Fisher Scientific, Waltham, MA, USA) in a ChemiDoc imaging system (Bio-Rad, Hercules, CA, USA) using ImageLab v5.2 software. The GAPDH (CB1001 from Calbiochem, Burlington, MA, USA) and Histone H3 (ab17684 from Abcam, Cambridge, UK) antibodies were employed as loading controls for the cytoplasmic and nuclear fractions, respectively. Finally, the intensity of bands was quantitated by densitometric analysis using the ImageJ v1.50i program.

### 4.5. Cell Proliferation and Invasion

To evaluate the proliferative ability of CC cell lines, C-33A, SiHa and HeLa cells were seeded (1 × 10^4^ cells/well) into 400 µL of medium in an E-plate L8 device (iCELLigence system, ACEA Biosciences, San Diego, CA, USA), after measuring the background in 100 µL of medium. Two replicates for each condition were analyzed. Cell attachment, spreading and proliferation were monitored by real-time cell analysis for 7 days, on the basis of changes in cell-sensor impedance, as previously described [47]. To assess the invasive capability of CC cell lines, cells were starved overnight. Next day, extracellular matrix layer was prepared by adding 40 µL of Matrigel^®^ (BD Biosciences, Franklin Lake, NJ, USA) into Transwell^®^ inserts with 8-μm-pore membranes (Sarstedt, Nümbrecht, Germany). After gelling for 15–30 min at 37 °C, starved cells were seeded (1.25 × 10^5^ cells/insert) on the gel layer in FBS-free medium, and the insert was placed in a well with FBS-containing medium. Cells were allowed to digest and penetrate the Matrigel^®^ layer as far as the membrane for 72 h. Then, non-invading cells and the gel layer were removed, and membranes were fixed and stained with a crystal violet solution containing paraformaldehyde (Sigma-Aldrich, St Louis, MO, USA). Images were taken with a Leica DMi1 microscope and the Leica Application Suite v4.12 program (Leica, Wetzlar, Germany).

### 4.6. p16 Native form Identification

In order to explore whether p16 subcellular location depended on the 3D conformation of the p16+interactors complex, the p16 native form was interrogated in SCCC cell lines. To do this, C-33A and SiHa cells were scraped and centrifuged at 4000 rpm for 1 min. Pellets were resuspended in 50–200 µL of non-denaturing lysis buffer (20 mM Tris-HCl, 137 mM NaCl, 2 mM EDTA and protease inhibitors) and incubated on ice for 5 min. After centrifuging at 8000× *g* for 10 min at 4 °C, proteins contained in the supernatants were quantified, and 50 µg of native protein Were subjected to PAGE on an 8% polyacrylamide gel under non-denaturing conditions. Transfer, blocking, incubation with antibodies and revealing were performed as described above.

### 4.7. p16 Immunoprecipitation

To identify cytoplasmic p16 interactors, p16 was first immunoprecipitated from SiHa cells. Briefly, cells were grown to 70–80% confluence, lysed with a home-made IP buffer (50 mM Tris-HCl, 150 mM NaCl, 1 mM EDTA, 1% Triton and protease inhibitors) and centrifuged at maximum speed for 10 min. Proteins contained in the supernatants were quantified as described above, and three aliquots were separated: 50 µg Were denatured and stored to check the input by western blot; 5 mg Were incubated with 10 µg of anti-p16 antibody (ab108349, Abcam, Cambridge, UK); and 5 mg Were incubated with an irrelevant antibody (rabbit IgG, 10500C, Thermo Fisher Scientific, Waltham, MA, USA). Incubations were performed overnight at 4 °C in a rotating wheel. The next day, 30 µL of protein A/G-coated magnetic beads (Thermo Fisher Scientific, Waltham, MA, USA) Were added to each sample and incubated for 2 h at room temperature to allow them to bind to the antigen-antibody complex. Extracts were sequentially washed three times with IP buffer using a magnetic stand. Finally, 30 µL of IP buffer and 10 µL of Laemmli buffer 4× Were added, and samples were heated at 95 °C for 5 min to denature and unbind the magnetic beads. A magnetic stand was used to remove magnetic beads. A small 5-µL fraction of the eluted extract was separated to check immunoprecipitation efficiency by western blot, using the α-tubulin antibody (T-6074 from Sigma-Aldrich, St Louis, MO, USA) as a loading control, while 15 µL Were subjected to mass spectrometry.

### 4.8. Mass Spectrometry and Bioinformatic Analysis

SiHa protein extracts incubated with the anti-p16 and the IgG antibodies were analyzed by LC-MS/MS using two parallel strategies: i) extracts were subjected to SDS-PAGE in a Criterion™ TGX Stain-Free™ protein gel (Bio-Rad, Hercules, CA, USA), and bands were individually excised from the gel and then digested; ii) extracts were directly digested in solution. In both cases, protein extracts were trypsin-digested and cleaned with ZipTip. Resulting samples were examined in a Triple-TOF 5600+ (Sciex, Framingham, MA, USA) coupled to a NanoLC Ultra 1D Plus nanochromatograph (Eksigent, Dublin, CA, USA), as previously described [48]. MS/MS data were acquired using Analyst 1.7.1 (Sciex, Framingham, MA, USA) and spectra files were processed with Protein Pilot Software v.5.0 (Sciex, Framingham, MA, USA) using the Paragon™ algorithm for database searching, Progroup™ for data grouping, and searched against the concatenated target-decoy UniProt human proteome reference database (Proteome ID: UP000005640, 73045 proteins, April 2018) plus Human papillomavirus type 16 database (Proteome ID: UP000009251, 9 proteins, April 2018). The FDR was estimated using a non-linear fitting method and the results displayed were those for a global FDR of 1% or better. Proteins unequivocally detected in both workflows in the extract with the anti-p16 antibody but not present in the anti-IgG sample and with an FDR < 1% were considered to be potential p16 interactors. Their biological functions were interrogated using the UniprotKB/Swiss-Prot (https://www.uniprot.org/) database. Additionally, the p16 interactors identified here were compared with those available in the interaction repository BioGRID (Biological General Repository for Interaction Datasets, v3.5.173, https://thebiogrid.org/) [49].

### 4.9. Immunofluorescence

In order to confirm p16 expression pattern in cultured SCCC cells and to assess colocalization of cytoplasmic p16 and its potential interactors, cells were seeded on coverslips and allowed to attach overnight. Cells were then fixed with 4% paraformaldehyde, blocked with 5% FBS in PBS at room temperature for 1 h, and incubated at 4 °C overnight with primary antibodies: anti-p16 (805-4713, Roche, Basel, Switzerland) at 250 ng/mL, and anti-CDK4 (ab108357, Abcam, Cambridge, UK), anti-BANF1 (CSB-PA002550LA01HU), anti-AKAP8 (STJ91533) or anti-AGTRAP (CSB-PA744194LA01HU) (all from Antibodyplus, Inc., Brookline, MA, USA), at 1:150. All samples were simultaneously incubated with AlexaFluor 488 anti-mouse and AlexaFluor 568 anti-rabbit (1:200) (Life Technologies, Carlsbad, CA, USA) at room temperature for 1 h. Finally, samples were mounted on slides with DAPI to counterstain nuclei. Images were acquired with a Leica DM4000B microscope (Leica, Wetzlar, Germany), using the NIS Elements v4.30 program (Nikon Instruments, Amsterdam, Netherlands), and excitation/emission wavelengths of 340–380/425 nm, 450–490/525 nm, and 560/40–645/75 nm for blue, green and red, respectively.

### 4.10. Statistical Analysis

Demographic, clinical and pathological data were summarized as frequencies (and percentages) and means (and ranges) ± standard error of the mean, as appropriate. All statistical analyses were carried out using IBM SPSS Statistics v25. Immunohistochemical expression levels in HSIL and SCCC samples, as well as differences in cell line proliferation, were compared using the Mann-Whitney test. Association between clinical variables and p16 expression or location was tested using a χ^2^ contingency test. Kaplan-Meier plots and log-rank tests were used to examine the association of p16 expression and location, age, stage and vascular invasion with PFS and OS in SCCC patients. A multivariate Cox regression model was fitted to test the independent contribution of each variable to the patient’s outcome after adjusting. Hazard ratios and 95% confidence intervals were used to estimate the effect of each variable on the outcome. 

## 5. Conclusions

In conclusion, we have gained further insight into the paradox of p16 overexpression in CC: it is expressed at high levels, but not located where it would act as a tumor-suppressor protein. We propose then that exploring p16 subcellular location can provide more useful information regarding anti-tumor activity than p16 overall expression examination. In particular, cytoplasmic p16, which is sequestered by novel proteins identified here for the first time, is more frequently observed in SCCC than HSIL and is associated with a worse outcome in SCCC. Therefore, the ratio of cytoplasmic to nuclear p16 and the absence of nuclear p16 could be independent biomarkers for SCCC diagnosis and prognosis, respectively.

## Figures and Tables

**Table 1:** Distribution of p16 expression and subcellular location in our series of 78 cervical tumors. Number and percentage of high-grade squamous intraepithelial lesions (HSILs) (*n* = 29) and squamous cell carcinomas of the cervix (SCCCs) (*n* = 49) patients with negative (0), weak (1), moderate (2) and strong (3) immunohistochemical expression of global, nuclear and cytoplasmic p16 protein levels. The cytoplasmic/nuclear p16 ratio was calculated by dividing cytoplasmic by nuclear p16 immunohistochemical score.

Global p16	No. of HSILs	No. of SCCCs
0	1 (3%)	2 (4%)
1	2 (7%)	3 (6%)
2	17 (59%)	6 (12%)
3	9 (31%)	38 (78%)
**Nuclear p16**		
0	1 (3%)	3 (6%)
1	4 (14%)	3 (6%)
2	10 (34%)	16 (33%)
3	14 (48%)	27 (55%)
**Cytoplasmic p16**		
0	1 (3%)	3 (6%)
1	8 (28%)	3 (6%)
2	19 (66%)	8 (16%)
3	1 (3%)	35 (71%)
**Cyt/nuc p16 ratio**		
<1	17 (59%)	6 (12%)
=1	8 (26%)	21 (43%)
>1	4 (14%)	22 (45%)

**Table 2:** Cytoplasmic p16 interactome in SiHa cells. 16 proteins were simultaneously identified in two independent experiments through in-solution- and in-gel-digested extracts from immunoprecipitated p16 in the SiHa cell line (false-discovery rate (FDR) < 0.01).

Protein Name	UniProt ID	Gene Name	Brief Description
Cyclin-dependent kinase 4	P11802	*CDK4*	Ser/Thr-kinase which phosphorylates and inhibits members of the Rb protein family to allow dissociation of E2F, which is responsible for the progression through the G_1_ phase of the cell cycle.
Complement component 1 Q subcomponent-binding protein, mitochondrial	Q07021	*C1QBP*	Involved in inflammation and infection processes, ribosome biogenesis, protein synthesis in mitochondria, regulation of apoptosis, transcriptional regulation and pre-mRNA splicing. It is required for the nuclear translocation of splicing factor U2AF1L4. Involved in regulation of CDKN2A-mediated apoptosis. Stabilizes mitochondrial CDKN2A isoform smARF.
Type-1 angiotensin II receptor-associated protein	Q6RW13	*AGTRAP*	Negative regulator of type-1 angiotensin II receptor-mediated signaling.
A-kinase anchor protein 8	O43823	*AKAP8*	Anchoring protein which mediates the subcellular compartmentation of PKA type II. May help to deliver cyclin D/E to CDK4 to facilitate cell cycle progression. Involved in nuclear retention of RPS6KA1 upon ERK activation thus inducing cell proliferation. May be involved in recruitment of active CASP3 to the nucleus in apoptotic cells. May act as a carrier protein of GJA1 for its transport to the nucleus.
THO complex subunit 4	E9PB61	*ALYREF*	Export adapter involved in nuclear export of spliced and unspliced mRNA.
Barrier-to-autointegration factor	O75531	*BANF1*	Plays fundamental roles in nuclear assembly, chromatin organization, gene expression and gonad development. Promotes integration of viral DNA into the host chromosome.
ATP-dependent RNA helicase DDX24	Q9GZR7	*DDX24*	ATP-dependent RNA helicase.
40S ribosomal protein S30	E9PR30	*FAU*	Ubiquitin-like and ribosomal protein S30 fusion.
Heterogeneous nuclear ribonucleoprotein U-like protein 1	Q9BUJ2	*HNRNPUL1*	Represses transcription driven by several virus and cellular promoters.
Polyadenylate-binding protein 2	Q86U42	*PABPN1*	Involved in the 3′-end formation of mRNA precursors by the addition of a poly(A) tail and various stages of mRNA metabolism including nucleocytoplasmic trafficking.
cGMP-specific 3’,5’-cyclic phosphodiesterase	O76074	PDE5A	Hydrolysis of cGMP to 5′-GMP.
Pregnancy zone protein	P20742	*PZP*	Proteinase inhibition.
U5 small nuclear ribonucleoprotein 200 kDa helicase	O75643	*SNRNP200*	RNA helicase essential for pre-mRNA splicing.
Serine/arginine repetitive matrix protein 1	Q8IYB3	*SRRM1*	Part of pre- and post-splicing multiprotein mRNP complexes involved in numerous pre-mRNA processing events.
Testis-specific Y-encoded-like protein 2	Q9H2G4	*TSPYL2*	May inhibit cell proliferation by inducing p53-dependent *CDKN1A* expression.
ATPase WRNIP1	Q96S55	*WRNIP1*	Modulator of DNA polymerase delta-mediated DNA synthesis.

## Supplementary Materials

Supplementary materials can be found at https://www.mdpi.com/1422-0067/21/6/2125/s1.

## Abbreviations

AGTRAP	Type-1 angiotensin II receptor-associated protein
AKAP8	A-kinase anchor protein 8
ATCC	American Type Cell Collection
BANF1	Barrier to autointegration factor
BioGRID	Biological General Repository for Interaction Datasets
C1QBP	Complement component 1 Q subcomponent-binding protein
CC	Cervical cancer
CDK4	Cyclin-dependent kinase 4
CI	Confidence interval
FBS	Fetal bovine serum
FDR	False-discovery rate
HR-HPV	High-risk human papillomavirus
HSIL	High-grade squamous intraepithelial lesion
LC-MS/MS	Liquid chromatography-tandem mass spectrometry
OS	Overall survival
p16	Cyclin-dependent kinase inhibitor 2A protein
PBS	Phosphate-buffered saline
PFS	Progression-free survival
Rb	Retinoblastoma protein
SCCC	Squamous cell carcinoma of the cervix
