# Supplementary material for: Absence of Nuclear p16 Is a Diagnostic and Independent Prognostic Biomarker in Squamous Cell Carcinoma of the Cervix
Source: Int J Mol Sci. 2020 Mar 19;21(6):2125. doi: 10.3390/ijms21062125 (PMC7139571; doi:10.3390/ijms21062125)
Supplement: Supplementary file 1 [file ijms-21-02125-s001.zip › supplementary/Mendaza et al_p16 in SCCC_Supplementary Information.docx]

**Supplementary Information**

The Supplementary Material contains three tables and three figures.

**Supplementary Tables**

**Supplementary Table S1.** Pathological and clinical parameters of the four SCCC patients in our series with no or low p16 expression. Three of them (IDs 7, 33 and 38) lacked nuclear expression of p16.

| Patient ID | **Age (y)** | **Grade** | **LNI** | **VI** | **Stage** | **HR-HPV status** | **Progression** | **PFS (m)** | **Exitus** | **OS (m)** | **FU (m)** | **Status at last FU** |
| --- | --- | --- | --- | --- | --- | --- | --- | --- | --- | --- | --- | --- |
|  |  |  |  |  |  |  |  |  |  |  |  |  |
| 7 | 58 | 1 | Yes | Yes | IVB | Positive | Yes | 3 | Yes | 5 | 5 | Deceased |
| 33 | 32 | 1 | No | Yes | IB1 | Positive | No | 159 | No | 159 | 159 | AWOD |
| 38 | 68 | 3 | No | Yes | IIA | Positive | Yes | 11 | Yes | 17 | 17 | Deceased |
| 50 | 72 | 3 | Yes | Yes | IIIB | Positive | Yes | 10 | Yes | 23 | 23 | Deceased |

y: years; LNI: lymph node involvement; VI: vascular invasion; m: months; FU: follow-up; AWOD: alive without disease

**Supplementary Table S2. Protein identification by LC-MS/MS.** Proteins that immunoprecipitated with the anti-p16 antibody and the irrelevant IgG antibody were in-gel-and in-solution-digested and subjected to LC-MS/MS.

**Supplementary Table S3.** Pathological and clinical characteristics of our series of 49 SCCC patients (NA, not available).

| **Feature** | | **Frequency (%)** |
| --- | --- | --- |
| **Age** (years) | Mean:  Range: | 52  30-82 |
| **Grade** | |  |
| 1 | | 12 (24.5) |
| 2 | | 15 (30.6) |
| 3 | | 13 (26.5) |
| NA | | 9 (18.4) |
| **Lymph node involvement** | |  |
| No | | 33 (67.3) |
| Yes | | 12 (24.5) |
| NA | | 4 (8.2) |
| **Vascular invasion** | |  |
| No | | 20 (40.8) |
| Yes | | 23 (46.9) |
| NA | | 6 (12.2) |
| **Stage** | |  |
| I | | 23 (46.9) |
| II | | 7 (14.3) |
| III | | 8 (16.3) |
| IV | | 6 (12.2) |
| NA | | 5 (10.2) |
| **HR-HPV status** | |  |
| Negative | | 0 (0.0) |
| Positive | | 49 (100.0) |
| **Progression-free survival** (months) | Mean:  Range: | 114  3-237 |
| Not relapsed | | 29 (59.2) |
| Relapsed | | 16 (32.7) |
| NA | | 4 (8.2) |
| **Overall survival** (months) | Mean:  Range: | 127  5-237 |
| Alive | | 32 (65.3) |
| Exitus | | 13 (26.5) |
| NA | | 4 (8.2) |
| **Therapy** | |  |
| Only chemotherapy | | 0 (0.0) |
| Only radiotherapy | | 9 (18.4) |
| Chemotherapy+radiotherapy | | 25 (51.0) |
| NA | | 15 (30.6) |

**Supplementary Figures**

**
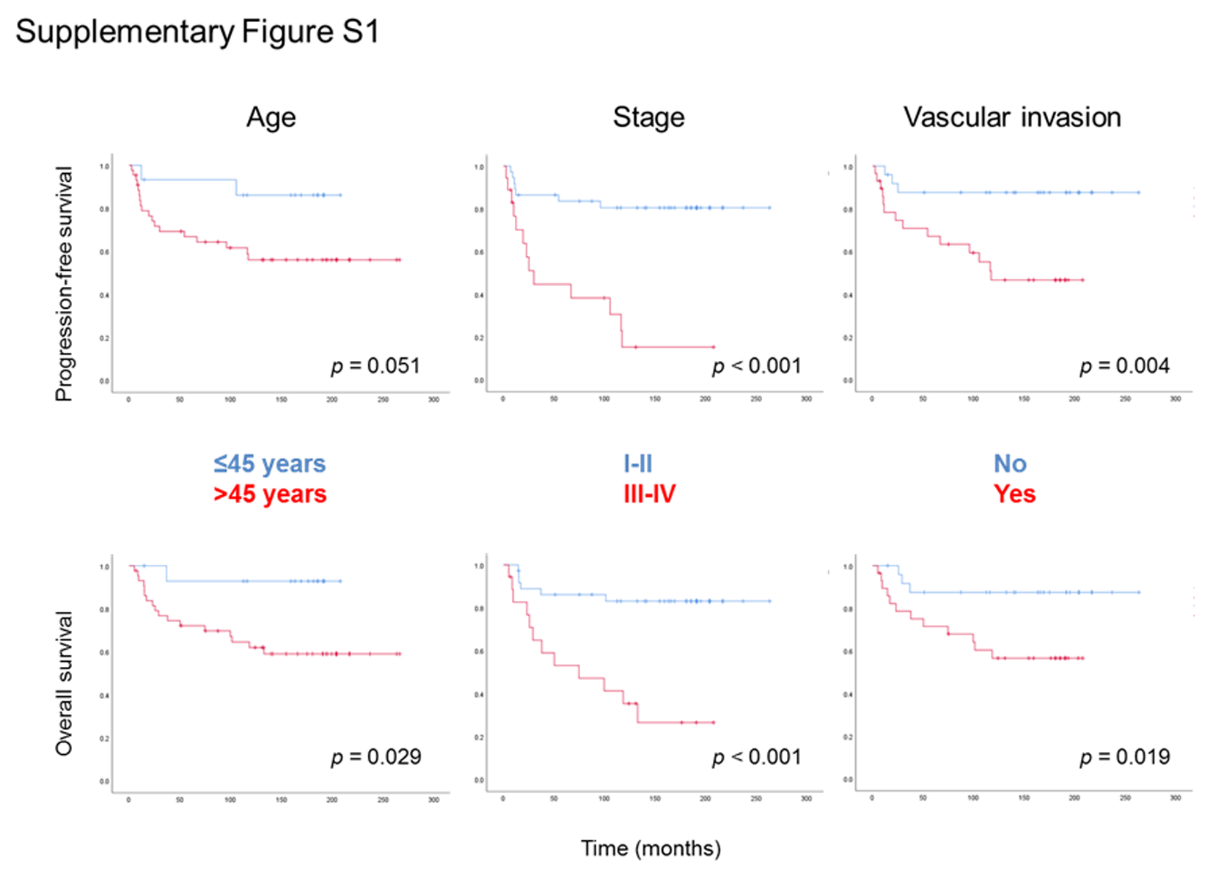
**

**Supplementary Figure S1. Relevant clinical parameters in SCCC prognosis and outcome.** Variables such as age, stage and vascular invasion were significantly associated with either PFS or OS in our SCCC patient series.


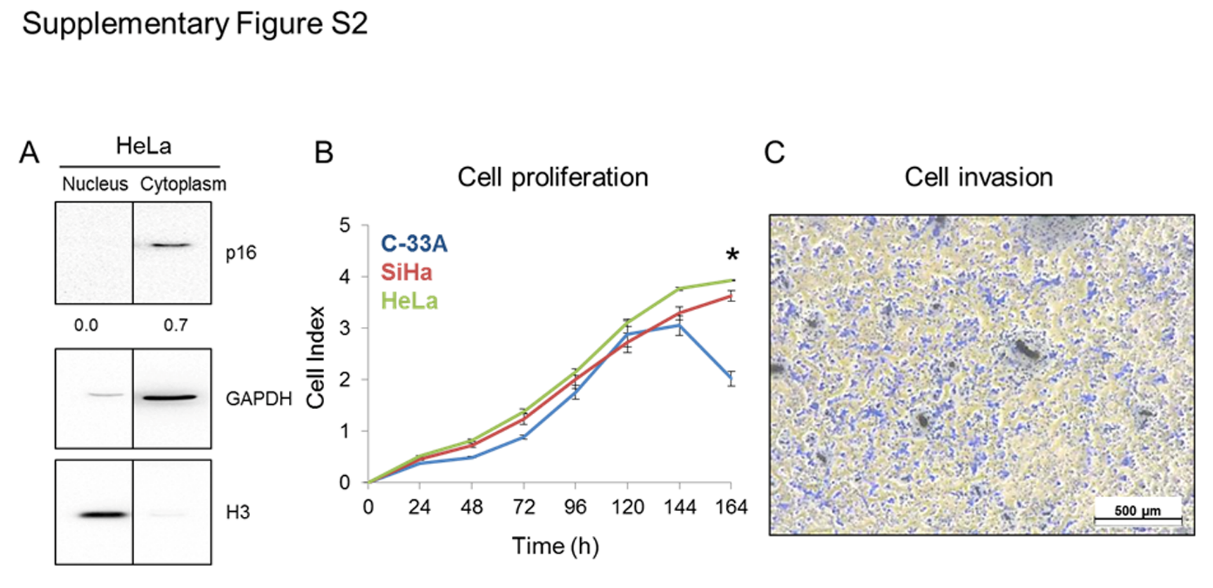


**Supplementary Figure S2. Subcellular location of p16 and aggressive properties of the HeLa cell line. (a)** Cell compartments of HeLa cells were fractionated and p16 expression was checked in nucleus and cytoplasm by western blot, using Histone H3 and GAPDH as loading controls, respectively. Numbers indicate the ratio of p16 signal relative to that of the loading control, measured by densitometry. **(b)** HeLa cell proliferation was measured by real-time cell analysis for 7 days and compared with that of the C-33A and SiHa cell lines (*, *p*<0.05). **(c)** HeLa cell invasion was measured as the ability to pass through a Matrigel® layer for 3 days. Images were acquired at 50X magnification.

**
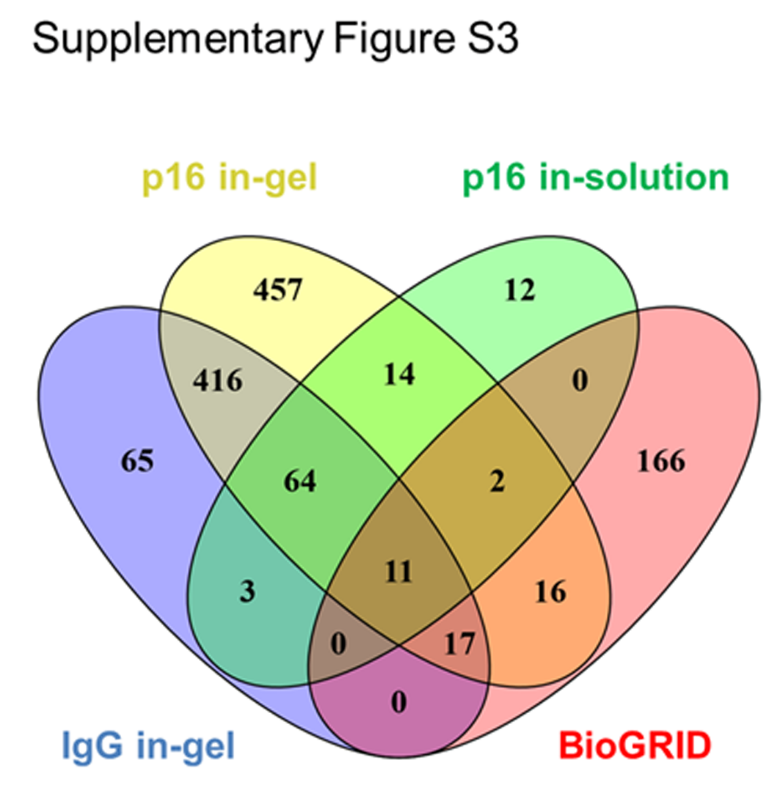
**

**Supplementary Figure S3. Cytoplasmic p16 interactome in SiHa cells.** Venn diagram showing the number of proteins identified by LC-MS/MS for each condition: in-gel-digested IgG IP extract, in-gel-digested p16 IP extract, and in-solution-digested p16 IP extract, along with p16 interactors currently available from the BioGRID repository. The only two proteins common to the three lists of interactors were CDK4 and C1QBP. To aid data interpretation, only in-gel digestion from the IgG extract is shown. Complete information, including the in-solution-digested IgG extract, is shown in Supplementary Table S2.
